# Prognostic Value of Radiotherapy and Chemotherapy in Stage I–III Merkel Cell Carcinoma

**DOI:** 10.3389/fmed.2022.845905

**Published:** 2022-02-18

**Authors:** Aihong Bi, Sifu Yang, Yang Ding, Yong Yu, Wenming Zhan, Tao Song

**Affiliations:** ^1^Department of Radiation Oncology, Cancer Center, Zhejiang Provincial People's Hospital, Affiliated People's Hospital, Hangzhou Medical College, Hangzhou, China; ^2^Department of Medical Oncology, Cancer Center, Zhejiang Provincial People's Hospital, Affiliated People's Hospital, Hangzhou Medical College, Hangzhou, China; ^3^Department of Dermatology, Plastic Surgery Center, Zhejiang Provincial People's Hospital, Affiliated People's Hospital, Hangzhou Medical College, Hangzhou, China

**Keywords:** merkel cell carcinoma, SEER, radiotherapy, chemotherapy, survival, prognostic factor

## Abstract

**Purpose:**

Merkel cell carcinoma (MCC) is a highly malignant cancer associated with dismal survival outcomes. Surgery is the cornerstone for the management of MCC, but the benefit of radiotherapy (RT) and chemotherapy (CT) is still controversial. We aimed to investigate the prognostic value of RT and CT in the management of stage I-III MCC patients using the Surveillance, Epidemiology, and End Results (SEER) database.

**Methods:**

Patients with a histopathological diagnosis of MCC between 2010 and 2016 were included. The primary endpoint of this study was overall survival (OS). The prognostic significance for OS was analyzed by Cox proportional hazard regression model.

**Results:**

A total of 1,691 patients were identified in the SEER database. Over half of the patients had received RT (56.7%), and 9.8% of the patients were documented to have received CT. The median OS for the entire cohort was 66.0 months, and the 5-year OS rate was 53.8%. In the multivariate analysis, receiving RT was associated with significantly improved OS (*P* < 0.001), while receiving CT significantly negatively impacted OS (*P* = 0.010). In stage III patients who underwent treatment based on surgical resection, RT was still demonstrated to be a positive factor (*P* = 0.002), while CT had no significant association with OS in the univariate analysis (*P* = 0.295).

**Conclusions:**

The current data in the SEER database are consistent with earlier studies supporting the benefit of adjuvant RT for stage I-III MCC patients, but caution should be taken regarding the routine use of CT. For stage III MCC patients, the value of adjuvant CT needs to be confirmed in future studies.

## Introduction

Merkel cell carcinoma (MCC) is a rare but aggressive form of cancer that was first described in 1972 by Cyril Toker (1). Advanced age at diagnosis, excessive exposure to ultraviolet radiation, Caucasian race and infection with Merkel cell polyomavirus have already been characterized as significant risk factors for the incidence of MCC (2–4). Because of its rarity, high-level evidence originating from phase III, randomized controlled trials for the management of MCC over the past several decades is still lacking (5).

Traditionally, local surgery has played a critical role in the management of stage I-III MCC, in accordance with accumulating evidence supporting the sentinel lymph node biopsy (SLNB) procedure for MCC patients who could tolerate surgical excision. In addition, radiotherapy (RT) and/or chemotherapy (CT) have also been explored as adjuvant therapeutic strategies (6). A retrospective study of 1,665 cases of MCC from the Surveillance, Epidemiology, and End Results (SEER) program between 1973 and 2002 suggested that MCC patients receiving adjuvant RT had improved overall survival (OS, 63 months for the adjuvant RT group vs. 45 months for patients who did not receive RT, *P* = 0.0002) (7). In addition, the efficiency of CT in combination with RT or alone has also been evaluated in earlier studies and demonstrated partial survival benefits (8–11). In a pooled analysis of locally advanced or metastatic MCC patients who received first-line CT (8), the objective response rate (ORR) was 69%, with a median OS time of 24 months for locally advanced MCC patients. However, considering the increased incidence of treatment-related sequelae, immune suppression and decreased quality of life (QoL), CT is recommended only with caution in indicated high-risk patients based on a clinical assessment.

The National Cancer Institute's SEER database contains cancer diagnosis, treatment and survival data for nearly 30% of the U.S. population and has been demonstrated to have unique advantages in cancer research (12). Given this context, we designed the current study to further explore the prognostic influence of RT and CT on OS in stage I-III MCC patients in the contemporary era.

## Methods

### Patient Selection and Data Collection

**Figure 1:**
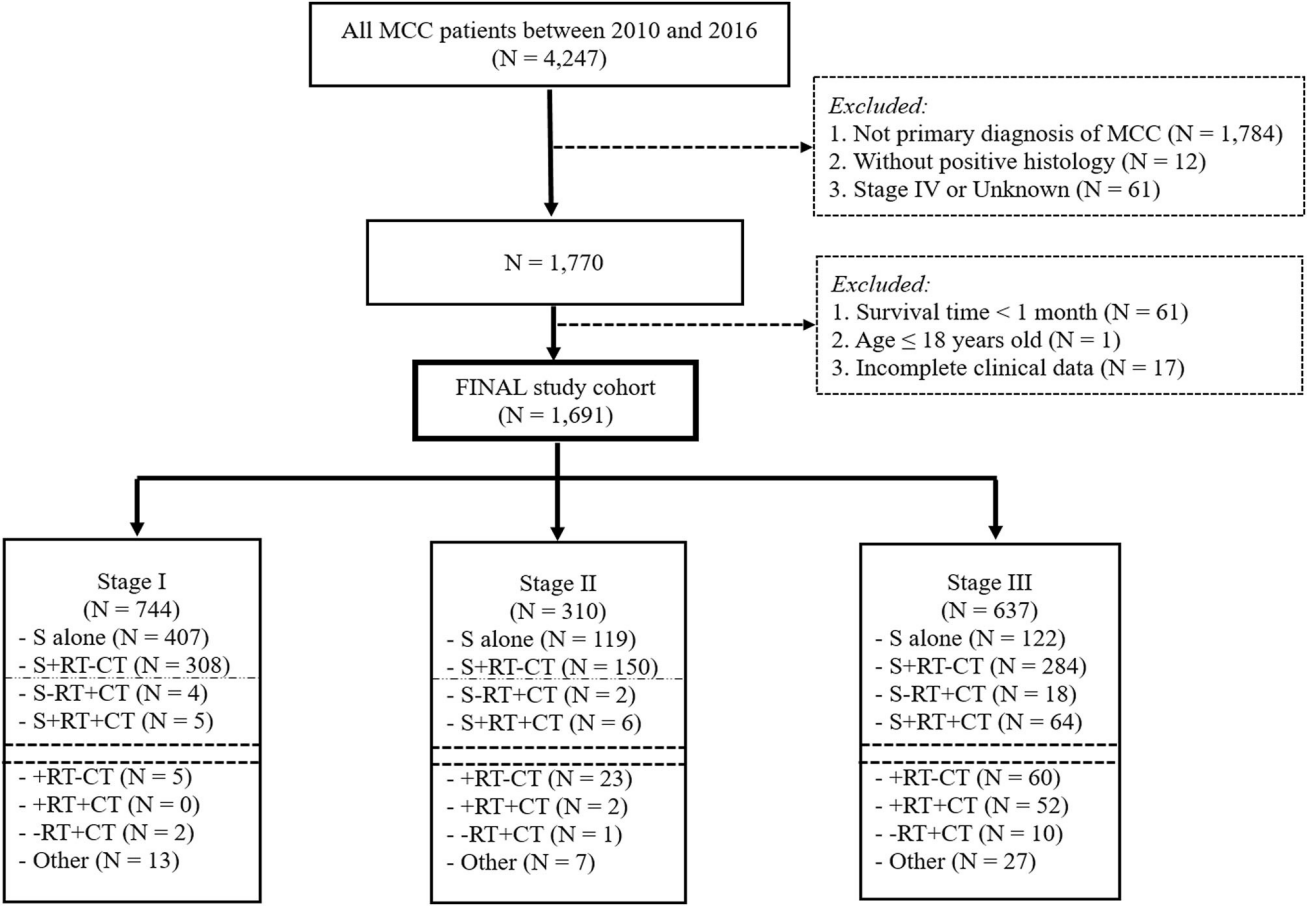
Patient selection flowchart.

The ICD-O-3 morphology code 8,247/3 was used to extract MCC (the ICD-O-3 code 8,247/2 was abandoned because no information was present under this item). The major inclusion criteria were as follows: (1) patients with a histopathological diagnosis of MCC from 2010 to 2016; and (2) patients over 18 years old with a primary diagnosis of MCC. The exclusion criteria were as follows: (1) records of other cancers and for whom MCC was not the first diagnosed cancer; (2) patients diagnosed with stage IV or an unknown stage registered in the database; and (3) patients whose survival time was <1 month or who had missing data for clinicopathological characteristics (Figure 1).

Age at diagnosis, sex, race, tumor site, tumor stage (2010–2015) based on the AJCC 7th Edition, surgery data including surgery at the primary site and surgery of the regional lymph nodes, treatment with RT, treatment with CT, cause of death (COD), cause-specific death, other cause of death, survival time in months and survival status (alive or dead) were extracted from the program. For MCC patients registered in 2016, tumors were restaged based on the stage definitions in the AJCC 7th edition.

### Data Analysis

OS was defined as the primary endpoint based on the perspectives proposed for cancers with longer survival and was calculated as the duration from the diagnosis of MCC to any cause of death or the last follow-up registered in the SEER program (13, 14). Patients' baseline characteristics were summarized by descriptive statistics and frequency tables. A restricted cubic spline (RCS) with 4 knots (5th, 35th, 65th, and 95th centiles) was used to model the association between age at diagnosis and OS, which included data under the items of survival time in months and survival status (15, 16). The Spearman's correlation coefficient analysis was applied to demonstrate the correlation among different variables. OS analysis was conducted using the Kaplan-Meier method and further compared with the log-rank test. Variables with *P* ≤ 0.05 in univariate analysis were entered into a multivariate Cox regression model to identify independent prognostic factors of OS. Methods for univariate and multivariate analysis were also described in our previous studies (17, 18). Differences were considered significant if the two-sided *p*-values were <0.05.

All statistical analyses were conducted using R software, with the *rms, corrplot* and *survminer* packages in the R platform, version 3.6.2 (https://www.r-project.org) and the Statistical Product and Service Solutions (SPSS) version 25.0 (SPSS, Inc., Chicago, Illinois).

## Results

### Demographic and Baseline Characteristics

**Figure 2:**
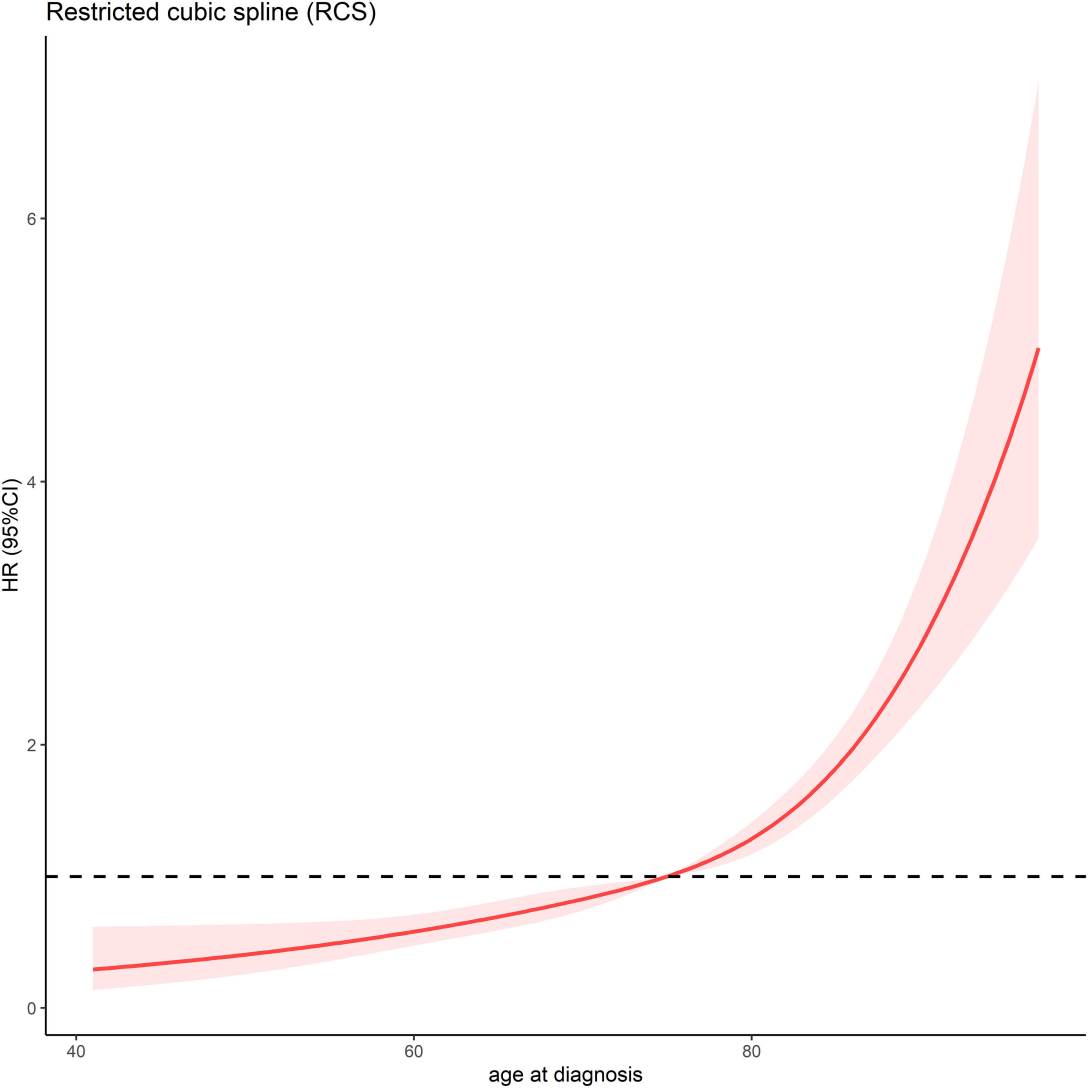
Association between age at diagnosis and OS time using a restricted cubic spline regression model. The dotted, black line represents the 1 HR and 95% CI for the spline model (reference is 75 years old).

**Figure 3:**
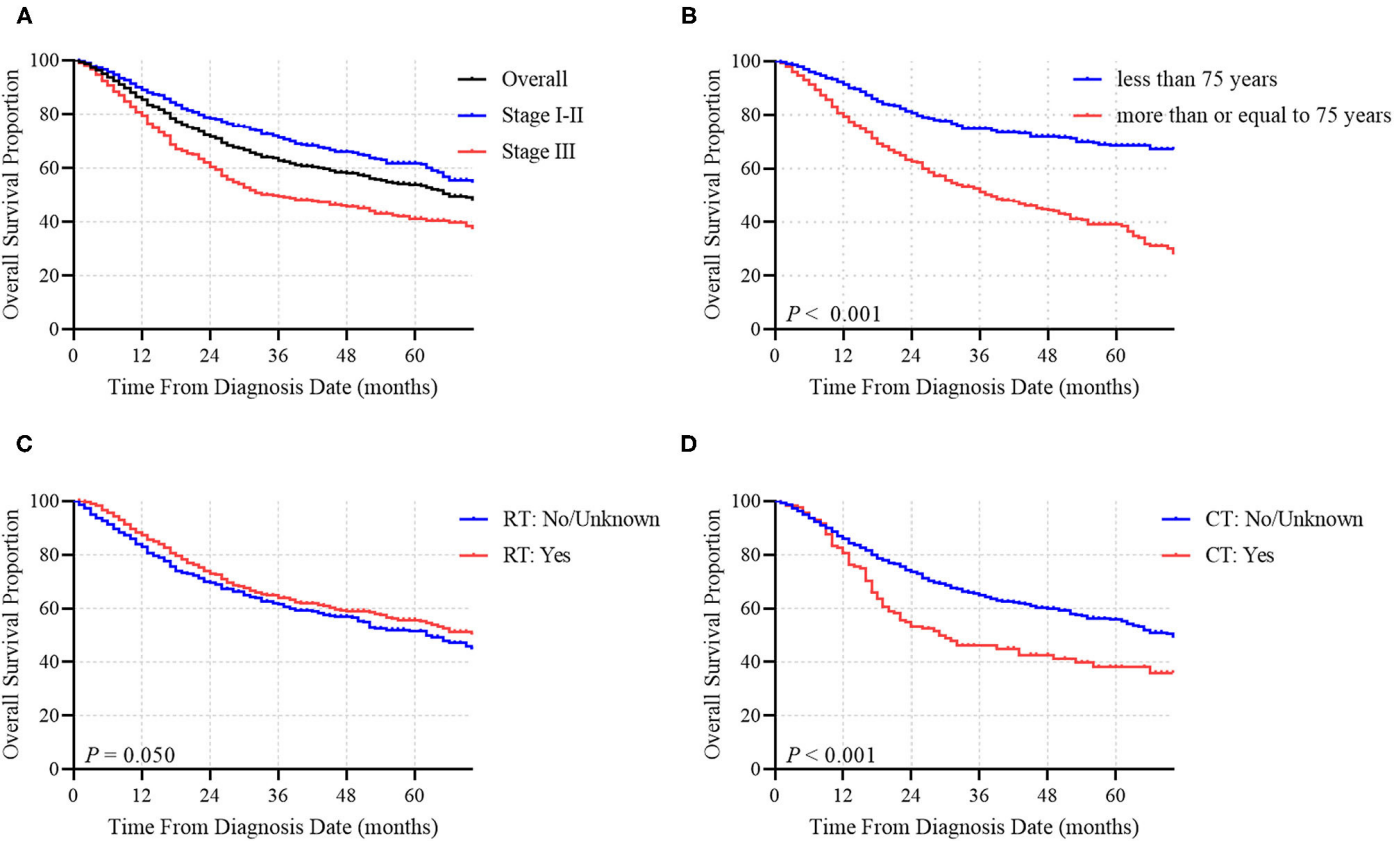
OS of MCC patients according to **(A)** tumor stage, **(B)** age at diagnosis, **(C)** RT or **(D)** CT.

In total, 4,247 MCC patients between 2010 and 2016 were extracted in the registry. Among these, 1,691 stage I-III MCC patients were included in the final analysis. The demographic, clinicopathological and treatment characteristics of these patients are presented in Table 1. 1,599 (94.6%) patients were white, and 1,020 (60.3%) patients were male. The most common tumor sites were the extremities (43.1%) and the head and neck (40.7%). The percentages of MCC patients with stage I, stage II and stage III disease were 44.0%, 18.3, and 37.7%, respectively. A total of 88.1% of the patients underwent cancer-directed surgery, and 1,133 (67.0%) MCC patients underwent SLNB and/or lymph node examination/removal. Overall, 959 (56.7%) patients had received RT; among these patients, 903 (94.2%) were documented to have postoperative RT, and 166 (9.8%) were assigned to receive CT. We then used RCS analysis to flexibly model the association between age at diagnosis and OS (Figure 2). Based on the result calculated by one hazard ratio (HR) and 95% confidence interval (CI), demonstrated in the dotted, black line, the appropriate inflection point to age at diagnosis was 75 years old. The OS comparison for younger (<75 yeas) and older (≥75 years) subgroup MCC patients is further presented in Figure 3B.

**Table 1:** Baseline characteristics of stage I-III MCC patients.

**Characteristic**	**Frequency, (%)**
*Age (years)*	
Median (IQR)	75 (66–83)
*Race*	
White	1,599 (94.6)
Non-white	92 (5.4)
*Sex*	
Female	671 (39.7)
Male	1,020 (60.3)
*Tumor location*	
Head and neck	689 (40.7)
Extremity	728 (43.1)
Trunk and others	274 (16.2)
*T stage*	
T_1−2_	1,441 (85.2)
T_3−4_	155 (9.2)
T_x_	95 (5.6)
*N stage*	
Negative	1,054 (62.3)
Positive	637 (37.7)
*Stage (AJCC 7th ed)*	
I	744 (44.0)
II	310 (18.3)
III	637 (37.7)
*Surgery at the primary site*	
No/unknown	202 (11.9)
Cancer-directed surgery performed	1,489 (88.1)
*SLNB and/or LN examination/removal*	
No/unknown	558 (33.0)
Yes	1,133 (67.0)
*Radiotherapy (RT)*	
No/unknown	732 (43.3)
Yes	959 (56.7)
*Chemotherapy (CT)*	
No/unknown	1,525 (90.2)
Yes	166 (9.8)

*IQR, interquartile range; SLNB, sentinel lymph node (LN) biopsy*.

### Prognostic Analysis

The median OS time of the whole cohort was 66.0 months. The 1-, 3-, and 5-year OS rates were 85.4, 62.9 and 53.8%, respectively (Figure 3A). Among patients who did not receive RT, the median OS time was 63.0 months, while among patients who received RT, the median OS time was 73.0 months, corresponding to a significantly different OS time between the two groups (*P* = 0.050; Figure 3C). Conversely, the median OS times were 29.0 months for patients who had received CT and 70.0 months for patients who did not receive CT (Figure 3D). CT was demonstrated to have a statistically negative impact on OS (*P* < 0.001).

In the univariate analysis (Table 2), age at diagnosis, race, sex, tumor site, T stage, N stage, tumor stage, surgery at the primary site, SLNB and/or LN examination/removal, RT and CT were significantly associated with OS. In the multivariate analysis, the significant covariates were age at diagnosis (*P* < 0.001, HR = 2.486), race (*P* = 0.013, HR = 0.540), sex (*P* < 0.001, HR = 1.480), T stage (T_1−2_ vs. T_3−4_, *P* = 0.034, HR = 1.329; T_1−2_ vs. T_x_, *P* = 0.003, HR = 0.565), tumor stage (*P* < 0.001, HR = 2.890), surgery at the primary site (*P* = 0.048, HR = 0.770), SLNB and/or LN examination/removal (*P* < 0.001, HR = 0.517), RT (*P* < 0.001, HR = 0.706), and CT (*P* = 0.010, HR = 1.433). N stage was excluded in the multivariate analysis due to its high correlation with tumor stage (Supplementary Figure 1).

**Table 2:** Univariate and multivariate analyses of OS in stage I-III MCC patients.

	**Overall survival (OS)**							
	**Univariate**				**Multivariate**			
***Factor***	***P-value***	***HR***	***95% CI Lower***	***95% CI Upper***	***P-value***	***HR***	***95% CI Lower***	***95% CI Upper***
*Age, <75 vs. ≥ 75*	<0.001	2.485	2.079	2.972	<0.001	2.486	2.044	3.024
*Race, White vs. Non-white*	0.009	0.523	0.323	0.848	0.013	0.540	0.331	0.880
*Sex, Female vs. Male*	<0.001	1.510	1.262	1.807	<0.001	1.480	1.233	1.777
*Site, reference: Head & Neck*	<0.001				0.289			
Extremity	<0.001	0.677	0.563	0.815	0.224	0.888	0.733	1.076
Trunk and skin, NOS	0.181	0.851	0.672	1.078	0.168	0.829	0.635	1.082
*T stage, reference: T_1−2_*	<0.001				<0.001			
T*_3−4_*	<0.001	1.788	1.389	2.301	0.034	1.329	1.021	1.728
T*_*x*_*	0.822	0.960	0.670	1.374	0.003	0.565	0.387	0.824
N stage, Negative vs. Positive^*^	–				–			
*Stage, Stage I-II vs. III*	<0.001	1.908	1.614	2.256	<0.001	2.890	2.321	3.598
*Surgery at the Prim Site, No/Unknown vs. Yes*	<0.001	0.626	0.500	0.785	0.048	0.770	0.594	0.998
*SLNB and/or LN examination/removal, No/Unknown vs. Yes*	<0.001	0.590	0.498	0.700	<0.001	0.517	0.421	0.635
*RT recode, No/Unknown vs. Yes*	0.050	0.847	0.716	1.000	<0.001	0.706	0.591	0.843
*CT recode, No/Unknown vs. Yes*	<0.001	1.648	1.301	2.087	0.010	1.433	1.091	1.882

*: *N stage was excluded in latter analysis due to its high correlation with tumor stage (Supplementary Figure 1)*.

### Subgroup Analysis Based on Stage and Treatment Modality

Given that tumor stage was also an independent prognostic factor in the Cox regression model, we further conducted subgroup analysis in stage I-II MCC patients. Figure 1 demonstrated that, among 1,054 stage I–II MCC patients, 526 (49.9%) received surgery alone, while 458 (43.5%) received surgery and RT without CT. Other treatment combinations are also listed in Figure 1. Subsequent multivariate analysis was performed among patients who received surgery alone or a combined treatment modality with surgery and RT. Several variables were significantly associated with OS (Supplementary Table 1): age at diagnosis (*P* < 0.001, HR = 3.314), sex (*P* < 0.001, HR = 1.801), T stage (T_1−2_ vs. T_3−4_, *P* = 0.014, HR = 1.790), SLNB and/or LN examination/removal (*P* < 0.001, HR = 0.539), and treatment modality (*P* = 0.026, HR = 0.737).

In stage III MCC patients who underwent at least surgical resection, the variables significantly correlated with OS were age at diagnosis (*P* < 0.001, HR = 1.611), SLNB and/or LN examination/removal (*P* < 0.001, HR = 0.476) and RT (*P* = 0.002, HR = 0.614) (Supplementary Table 2). CT was shown to be a non-significant prognostic indicator correlated with OS in the initial univariate analysis (*P* = 0.295, HR = 1.209).

## Discussion

Although advanced age at diagnosis is known to be an independent prognostic factor associated with worse survival, different median or mean values for age at diagnosis have been reported in the literature (7, 19–22). Results from the RCS analysis in the current study indicated that an age of 75 years was an appropriate inflection point for subsequent analysis (Figure 2), which was consistent with prior large sample size studies (23, 24). In the multivariate analysis, advanced age at diagnosis was further proven to be an independent risk factor for the whole cohort as well as in the subgroup analysis. Recently, Bleicher et al. retrospectively reviewed and reported the medical history of 102 MCC patients in the USA, and their results demonstrated that tumor stage (*P* < 0.01) and advanced age at diagnosis (*P* < 0.01) were both risk factors for tumor recurrence and decreased OS time (25). Additionally, as shown in our multivariate Cox regression analysis for all enrolled patients, the use of CT was significantly correlated with decreased OS (*P* = 0.010, HR = 1.433). CT was only a non-significant prognostic factor correlated with OS in the subgroup analysis for stage III patients (*P* = 0.295 in the univariate analysis). Along with aging, lethal comorbidities other than MCC might significantly impact the survival time and QoL of MCC patients. Previously, Allen et al. retrospectively reviewed the treatment experience at Memorial Sloan-Kettering Cancer Center for MCC patients diagnosed between 1970 and 2002 (19). In a subgroup analysis of node-positive (stage III) patients, 23 patients received adjuvant CT, and 53 patients did not receive adjuvant CT. No significant difference was demonstrated in disease-specific survival (DSS) between the two groups (*P* = 0.08). Similarly, Bhatia et al. also investigated the role of adjuvant RT and CT in a large cohort of MCC patients (N = 6,680) registered in the NCDB database between 1996 and 2008 (26). In multivariable analyses, adjuvant CT was not observed to significantly improve OS in stage III patients (*P* = 0.71, HR = 0.97), consistent with the findings in the current SEER database analysis. Given the toxicities correlated with CT, a phase II Australian study first demonstrated that the combination of CT consisting of carboplatin and etoposide with concurrent RT appeared to show acceptable treatment-related toxicities for high-risk MCC patients (27). However, the final multivariate Cox analysis failed to show significant improvement in DSS for this treatment strategy compared with that for historic controls (28). Based on the above findings, CT with newer agents in node-positive or high-risk MCC patients should be investigated, but caution should be taken regarding the routine use of CT in the first-line therapy for the treatment of MCC.

Given the role of RT, a 2019 meta-analysis combining 29 studies of 17,179 MCC patients showed a significant difference in OS (*P* < 0.001, HR = 0.810) and disease-free survival (*P* < 0.001, HR = 0.450) in favor of adjuvant RT. Additionally, the meta-regression analysis further indicated that variables such as stage I-II tumors and a younger age at diagnosis were significantly associated with increased OS (29). Similar results supporting the benefit of adjuvant RT were also reported in another meta-analysis published in 2006 (30) and in the aforementioned NCDB study for stage I-II MCC patients (26). As for definitive RT, a previously published, retrospective analysis compared the efficiency between surgical resection and definitive RT in stage I-III MCC patients using data from NCDB (21). After employing propensity score matching to reduce imbalances between groups, surgical resection significantly improved OS in all stages. However, due to the lack of high-level evidence regarding the role of RT and a marked heterogeneity among MCC patients enrolled in different studies, the precise role of RT remains controversial and should be investigated in the future. The rationale for applying RT as an alternative therapy is that MCC is generally characterized as a neuroendocrine carcinoma, and that clinicians have limited choices beyond extrapolating MCC treatment options based on evidence from other types of neuroendocrine cancers, such as small cell lung cancer.

To date, accumulating evidence has shown encouraging results for immunotherapy in the treatment of MCC (23, 31). In 2016, Kaufman et al. conducted a single arm, phase II trial evaluating the efficiency of avelumab in CT-refractory metastatic MCC patients. Avelumab was found to be associated with durable responses, with an ORR of 31.8% among 88 patients. Treatment-related toxicity was well-tolerated (32). Subsequently, the use of first-line immunotherapy with avelumab in stage IV MCC patients was reported in 2018 (33). In this prospective trial, the ORR was 62.1%. Furthermore, the estimated percentage of responding patients with a response duration of ≥3 months was 93%, with no grade 4–5 treatment-related toxicities observed. In the Checkmate-358 trial (34). Thirty nine stage IIA-IV MCC patients were allocated to receive nivolumab monotherapy in the neoadjuvant setting followed by surgical resection. The results showed that among the 36 MCC patients who underwent subsequent focal surgery, 17 (47.2%) patients achieved a pathologic complete response (pCR). No patient with a pCR exhibited tumor relapse during the observation period, and a statistically significant difference in recurrence-free survival was shown between pCR and non-pCR patients (*P* = 0.043, HR = 0.12). However, the risks of using cancer immunotherapies should not be ignored. Recently, Roopkumar et al. retrospectively reviewed the medical results of 1,686 cancer patients who received immunotherapies and investigated the incidence of venous thromboembolism (VTE) and its impact on survival outcomes (35). The median age of the enrolled patients was 64.5 years. VTE was observed in 404 (24.0%) cancer patients. Survival analysis demonstrated that cancer patients who were diagnosed with VTE had a significantly decreased OS time compared with patients without VTE (365 days vs. 453 days; *P* = 0.002). In the multivariate analysis, a diagnosis of VTE was also indicated to be an independent prognostic factor for decreased survival (*P* = 0.008, HR = 1.220). Similar findings for the risks of VTE have also been reported in other studies (36, 37). In addition, attention should be paid to other immune-related adverse events, such as immunotherapy-related acute kidney injury (AKI). In a cohort of 13 cancer patients with AKI confirmed by kidney biopsy, the median time from the initiation of immunotherapy to AKI was only 91 days. Although the renal functions of 10 patients were recovered by receiving glucocorticoids, the effects of immunotherapy were certainly reduced (38).

This study is not without limitations. First, this study is a retrospective study. Second, some important information, such as patients' comorbidities, treatment complications, RT doses, CT regimens, sequences and cycles, and use of immunotherapy were not provided in the SEER dataset. Therefore, potential bias might have influenced the final results, and the findings, especially the value of adjuvant CT, need to be evaluated by well-designed large prospective studies in the future.

## Conclusions

The present study aimed to investigate the influence of demographic, clinical, and treatment factors on the OS outcomes of MCC patients registered in the SEER database between 2010 and 2016. In the final Cox regression model, factors including advanced age at diagnosis (≥75 years), white race, male sex, advanced tumor stage, lack of local surgical resection and SLNB and/or LN examination/removal, lack of treatment with RT, and treatment with CT were significantly correlated with decreased OS time. RT was demonstrated to be a positive prognostic factor in favor of increased OS, while CT, in contrast, was demonstrated to be a negative prognostic factor. Furthermore, subgroup analysis further supported the role of adjuvant RT in the treatment of MCC based on surgical resection. CT in the treatment of stage III MCC should be validated in the future, and comprehensive assessments should be made when balancing its efficacy and safety.

## Data Availability Statement

The original contributions presented in the study are included in the article/Supplementary Material, further inquiries can be directed to the corresponding author/s.

## Ethics Statement

Ethical review and approval was not required for the study on human participants in accordance with the local legislation and institutional requirements. Written informed consent for participation was not required for this study in accordance with the national legislation and the institutional requirements.

## Author Contributions

AB: conception and design, drafting, final approval, and accountable for aspects. SY: provision of study materials, collection and assembly of data, drafting, final approval, and accountable for aspects. YD: data analysis and interpretation, drafting, final approval, and accountable for aspects. YY: data analysis and interpretation, drafting, final approval, and accountable for aspects. WZ: conception and design, provision of study materials, collection and assembly of data, drafting, final approval, and accountable for aspects. TS: conception and design, provision of study materials, collection and assembly of data, drafting, final approval, and accountable for aspects. All authors contributed to the article and approved the submitted version.

## Conflict of Interest

The authors declare that the research was conducted in the absence of any commercial or financial relationships that could be construed as a potential conflict of interest.

## Publisher's Note

All claims expressed in this article are solely those of the authors and do not necessarily represent those of their affiliated organizations, or those of the publisher, the editors and the reviewers. Any product that may be evaluated in this article, or claim that may be made by its manufacturer, is not guaranteed or endorsed by the publisher.
