# Supplementary material for: Prognostic Value of Radiotherapy and Chemotherapy in Stage I–III Merkel Cell Carcinoma
Source: Front Med (Lausanne). 2022 Feb 18;9:845905. doi: 10.3389/fmed.2022.845905 (PMC8894769; doi:10.3389/fmed.2022.845905)
Supplement: Supplementary file 1 [file Table_1.docx]

Table S1. Univariate and multivariate analyses of OS in stage I-II MCC patients.

|  | Overall survival (OS) | | | | | | | | |
| --- | --- | --- | --- | --- | --- | --- | --- | --- | --- |
|  | Univariate | | | |  | Multivariate | | | |
| *Factor* | ***P*** *value* | *HR* | *95% CI Lower* | *95% CI Upper* |  | ***P*** *value* | *HR* | *95% CI Lower* | *95% CI Upper* |
| *Age, < 75 vs. ≥ 75* | < 0.001 | 4.130 | 3.019 | 5.648 |  | < 0.001 | 3.314 | 2.375 | 4.623 |
| *Race, White vs. Non-white* | 0.067 | 0.517 | 0.255 | 1.046 |  | - |  |  |  |
| *Sex, Female vs. Male* | < 0.001 | 1.645 | 1.255 | 2.156 |  | < 0.001 | 1.801 | 1.372 | 2.365 |
| *Site, reference: Head & Neck* | < 0.001 |  |  |  |  | 0.122 |  |  |  |
| Extremity | < 0.001 | 0.559 | 0.424 | 0.736 |  | 0.191 | 0.825 | 0.618 | 1.101 |
| Trunk and skin, NOS | 0.465 | 0.848 | 0.544 | 1.321 |  | 0.257 | 1.308 | 0.822 | 2.082 |
| *T stage, T1-2 vs. T3-4* | 0.022 | 1.685 | 1.077 | 2.639 |  | 0.014 | 1.790 | 1.125 | 2.849 |
| *SLNB and/or LN examination/removal, No/Unknown vs. Yes* | < 0.001 | 0.360 | 0.277 | 0.469 |  | < 0.001 | 0.539 | 0.405 | 0.717 |
| *Treatment modality, Surgery alone vs. S+RT* | 0.004 | 0.682 | 0.525 | 0.886 |  | 0.026 | 0.737 | 0.563 | 0.964 |
