# Supplementary material for: Prognostic Value of Radiotherapy and Chemotherapy in Stage I–III Merkel Cell Carcinoma
Source: Front Med (Lausanne). 2022 Feb 18;9:845905. doi: 10.3389/fmed.2022.845905 (PMC8894769; doi:10.3389/fmed.2022.845905)
Supplement: Supplementary file 2 [file Table_2.docx]

Table S2. Univariate and multivariate analyses of OS in stage III MCC patients.

|  | Overall survival (OS) | | | | | | | | |
| --- | --- | --- | --- | --- | --- | --- | --- | --- | --- |
|  | Univariate | | | |  | Multivariate | | | |
| *Factor* | ***P*** *value* | *HR* | *95% CI Lower* | *95% CI Upper* |  | ***P*** *value* | *HR* | *95% CI Lower* | *95% CI Upper* |
| *Age, < 75 vs. ≥ 75* | < 0.001 | 1.793 | 1.346 | 2.387 |  | 0.001 | 1.611 | 1.202 | 2.158 |
| *Race, White vs. Non-white* | 0.109 | 0.394 | 0.126 | 1.233 |  | - |  |  |  |
| *Sex, Female vs. Male* | 0.634 | 1.077 | 0.792 | 1.465 |  | - |  |  |  |
| *Site, reference: Head & Neck* | 0.103 |  |  |  |  | - |  |  |  |
| Extremity | 0.097 | 0.772 | 0.570 | 1.048 |  |  |  |  |  |
| Trunk and skin, NOS | 0.067 | 0.670 | 0.437 | 1.028 |  |  |  |  |  |
| *T stage*, T1-2 vs. T3-4* | 0.120 | 1.341 | 0.926 | 1.943 |  | - |  |  |  |
| *SLNB and/or LN examination/removal, No/Unknown vs. Yes* | < 0.001 | 0.442 | 0.295 | 0.661 |  | < 0.001 | 0.476 | 0.315 | 0.721 |
| *RT recode, No/Unknown vs. Yes* | 0.002 | 0.621 | 0.460 | 0.836 |  | 0.002 | 0.614 | 0.454 | 0.830 |
| *CT recode, No/Unknown vs. Yes* | 0.295 | 1.209 | 0.848 | 1.724 |  | - |  |  |  |

^*^: excluded 53 patients diagnosed with T_x_.
