# Supplementary figures and images for: Prognostic Value of Radiotherapy and Chemotherapy in Stage I–III Merkel Cell Carcinoma
Source: Front Med (Lausanne). 2022 Feb 18;9:845905. doi: 10.3389/fmed.2022.845905 (PMC8894769; doi:10.3389/fmed.2022.845905)

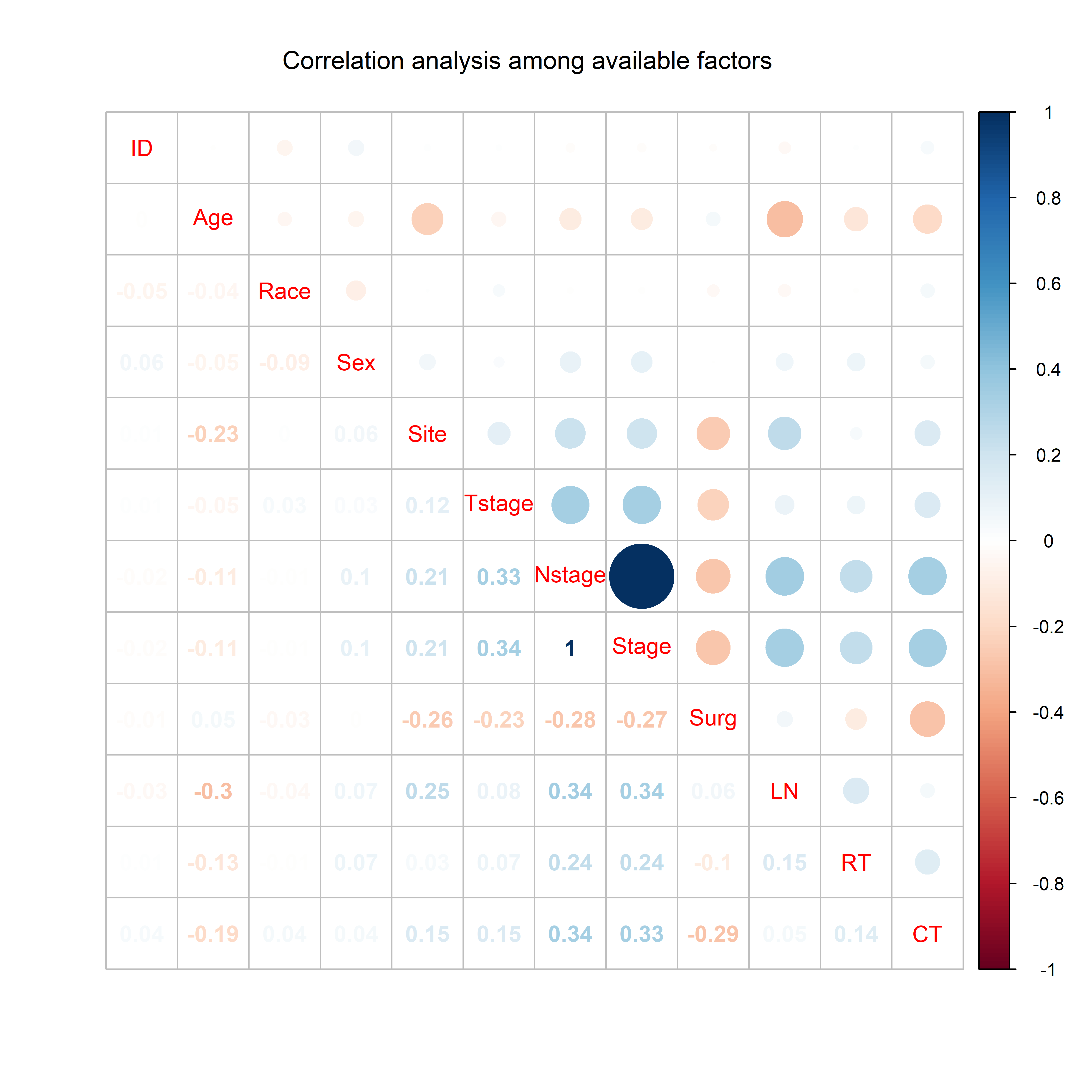

Supplement: Supplementary file 3 [file Image_1.TIF]
